# Correlation between TXNRD1/HO-1 expression and response to neoadjuvant chemoradiation therapy in patients with esophageal squamous cell carcinoma

**DOI:** 10.1007/s10388-021-00904-3

**Published:** 2022-01-08

**Authors:** Ryujiro Akaishi, Fumiyoshi Fujishima, Hirotaka Ishida, Junichi Tsunokake, Takuro Yamauchi, Yusuke Gokon, Shunsuke Ueki, Toshiaki Fukutomi, Hiroshi Okamoto, Kai Takaya, Chiaki Sato, Yusuke Taniyama, Tomohiro Nakamura, Naoki Nakaya, Takashi Kamei, Hironobu Sasano

**Affiliations:** 1 Department of Surgery, Tohoku University Graduate School of Medicine, Sendai, Japan; 2 Department of Pathology, Tohoku University Hospital, Sendai, Japan; 3 Department of Health Record Informatics Information Security, Tohoku Medical Megabank Organization, Tohoku University, Sendai, Japan; 4 Department of Preventive Medicine and Epidemiology, Tohoku Medical Megabank Organization, Tohoku University, Sendai, Japan

**Keywords:** Thioredoxin reductase 1, Heme oxygenase-1, Esophageal squamous cell carcinoma, Neoadjuvant therapy, Chemoradiotherapy

## Abstract

**Background:**

Thioredoxin reductase 1 (TXNRD1) and heme oxygenase-1 (HO-1) are both involved in the nuclear factor erythroid 2-related factor 2 (Nrf2) pathway and play key roles in antioxidant responses. In patients with esophageal squamous cell carcinoma (ESCC), the correlation between the expression of these two proteins and the therapeutic response to neoadjuvant chemoradiation therapy (NACRT), as well as the difference in their expression after chemoradiotherapy, remains unknown.

**Methods:**

Proteins involved in the Nrf2 pathway were immunolocalized in carcinoma cells in ESCC patients on NACRT with 5-fluorouracil and cisplatin, followed by esophagectomy. The 8-hydroxydeoxyguanosine (8-OHdG) levels were used to quantify reactive oxygen species. The changes in immunoreactivity before and after NACRT (Δ) were assessed.

**Results:**

Tumor reduction following NACRT was significantly attenuated in pre-therapeutic biopsy specimens associated with high HO-1 status. TXNRD1Δ, HO-1Δ, and 8-OHdGΔ were significantly different in the ineffective and effective groups. The overall survival was significantly lower in high Nrf2 and TXNRD1 groups. In addition, high TXNRD1 expression was an independent prognostic factor in the multivariate analysis of overall survival.

**Conclusions:**

The study findings indicate that HO-1 status in pre-therapeutic biopsy specimens could predict response to NACRT, and TXNRD1 status could predict overall survival of ESCC patients.

**Supplementary Information:**

The online version contains supplementary material available at 10.1007/s10388-021-00904-3.

## Introduction

Esophageal squamous cell carcinoma (ESCC) represents the majority of esophageal cancer cases in Japan and Asia [1]. Neoadjuvant chemotherapy (NAC) followed by surgical resection with lymph node dissection is commonly used to treat locally advanced ESCC [2]. However, strategies for treating patients with resistance to NAC are yet to be established [3, 4]. Clinical trials on neoadjuvant chemoradiation therapy (NACRT) using 5-fluorouracil/cisplatin with concurrent radiation therapy are underway for locally advanced ESCC [5–7] and may provide novel insights into optimal therapeutic approaches.

Reactive oxygen species (ROS) are known to mediate chemotherapy- or radiotherapy-induced damages to cancer cells. Nuclear factor erythroid 2-related factor 2 (Nrf2) is a transcription factor involved in the regulation of antioxidant protein expression in cells [8]. Nrf2 expression is enhanced in ESCC, resulting in the development of resistance to chemotherapy and radiotherapy [9]. Nrf2 also promotes the expression of antioxidant enzymes, including thioredoxin reductase 1 (TXNRD1) [10] and heme oxygenase-1 (HO-1) [11], which increases resistance to oxidative stress. TXNRD1 is one of the key enzymes that defend cancer cells against oxidative stress [10] and promotes cell proliferation and viability [12]. HO-1 is also an Nrf2 target, and its involvement in cell proliferation and development in cancer has been extensively studied [13, 14].

Low Nrf2 expression in pre-NACRT biopsy specimens was reported to be correlated with a favorable response to NACRT in ESCC patients [15]. However, the correlation between TXNRD1/HO-1 expression and response to NACRT has remained unknown. In addition, the prognostic significance of the proteins involved in these antioxidant pathways in human malignancies is yet to be established.

Therefore, in this study, we aimed to investigate the followings in ESCC patients: (1) predict NACRT efficacy and clinical outcomes/prognosis according to the status of TXNRD1/HO-1 in pre-NACRT endoscopic biopsy specimens and (2) examine the correlation between NACRT resistance and the difference of antioxidant protein expression in pre- and post-NACRT specimens.

## Materials and methods

### Patients and tumor specimens

The study enrolled 69 patients diagnosed with ESCC who underwent NACRT followed by thoracoscopic esophagectomy with regional lymph node dissection between 2011 and 2015 at Tohoku University Hospital, Sendai, Japan. Seventeen cases in which pre-therapeutic biopsy was not performed in our institution were tentatively excluded in this study. The tissue specimens available for comparison before and after NACRT were 39 cases (Supplementary Fig. 1). The criteria for therapeutic response to NACRT were tentatively determined as in Supplementary Table 1 [16]. Grade 0 or 1 response was interpreted as “ineffective,” whereas a Grade 2 or 3 response was considered “effective” [15].

TNM (tumor, nodes, and metastasis) staging was performed according to the guidelines defined in the eighth edition of the American Joint Committee on Cancer/Union for International Cancer Control [17]. The overall survival (OS) and recurrence-free survival (RFS) in patients were determined from the day NACRT commenced until death and recurrence, respectively, or based on the last follow-up.

The study protocol was approved by the Ethics Committee of the Tohoku University School of Medicine (Accession No. 2020-1-87), and informed consent was obtained from all participants prior to surgery.

### Neoadjuvant chemoradiation therapy

Chemotherapy was administered in conjunction with continuous intravenous infusion of 5-fluorouracil (400 mg/m^2^/day) for over 24 h on days 1–5 and 8–12 and with cisplatin (40 mg/m^2^) infusion for 2 h on days 1 and 8. Concurrent radiotherapy (total of 30 Gy in 15 fractions over 3 weeks) was performed.

### Immunohistochemistry

Immunohistochemical analyses were performed on formalin-fixed paraffin-embedded 4-micron-thick tissue sections. The immunohistochemical procedures are summarized in Supplementary Table 2. ROS levels in the tumor cells were evaluated using 8-hydroxydeoxyguanosine (8-OHdG) [18].

Each stained section was independently evaluated at the hot spots using 200 × magnification by two authors (RA and FF). Immunoreactivities of Nrf2 in the nuclei, TXNRD1, HO-1, and 8-OHdG in the cytoplasm were assessed semi-quantitatively using modified H-scores and by calculating the percentage of immunostained tumor cells multiplied by the relative immunointensity (0, negative; 1, weak; 2, moderate; 3, marked) [19]. Labeling index was applied for nuclear Ki-67 immunoreactivity evaluation [20]. The optimal cutoff values for the response of the patients were determined for pre-NACRT biopsies using the receiver operating characteristic curve (Supplementary Table 3) [19]. Differences in H-score and Ki-67 labeling index (Δ: post-NACRT − pre-NACRT values) were calculated.

### Statistical analysis

JMP® Pro version 14.2.0 (SAS Institute, Inc., Cary, NC, USA) was used for statistical analyses. Pearson’s chi-square tests, Fisher’s exact tests, Student’s *t*-tests, and Wilcoxon’s rank-sum tests were applied as appropriate. OS and RFS rates were investigated using the Kaplan–Meier method and compared using log-rank tests. The Cox proportional hazards model was used for univariate and multivariate analyses. Multivariate logistic regression analyses were also conducted. Variance inflation factor (VIF) among explanatory variables was calculated for each multivariate analysis. We confirmed that there was no multicollinearity in each multivariate analysis and VIF for each variable was ≤ 5. *P* < 0.05 was considered significant.

## Results

### Clinicopathological features of ESCC patients

The clinical characteristics of 52 cases are summarized in Table 1. Among these, the samples of 25 patients were tentatively classified as ineffective (Grade 0 or 1), and the remaining 27 patients exhibited a Grade 2 or 3 response to NACRT upon pathological analysis of the resected specimens. There were significant differences in the clinical (c) Stage between the ineffective and effective groups.

**Table 1:** Clinical features of the patients

Clinical features	*N*	Ineffective (Grade 0–1b)	Effective (Grade 2–3)	*P* value
		25	27	
Age ≥ 65	30	15	15	0.746
Age < 65	22	10	12	
Male	41	18	23	0.317
Female	11	7	4	
Smoker	38	18	20	0.866
Non-smoker	14	7	7	
Main location				0.488
Upper/Middle thoracic	35	18	17	
Lower thoracic/Esophagogastric junction	17	7	10	
cT^a^				0.069
cT1/2	15	4	11	
cT3/T4a	37	21	16	
cN^a^				0.055
cN0	13	3	10	
cN1/2	39	22	17	
Clinical stage^a^				0.005*
Stage I/II	23	6	17	
Stage III/IV	29	19	10	

*Statistical significance

^a^Tumor–node–metastasis (TNM) classification based on the 8th edition of the TNM classification of malignant tumors

### Expression status of antioxidant proteins and its correlation with response to NACRT

**Fig. 1:**
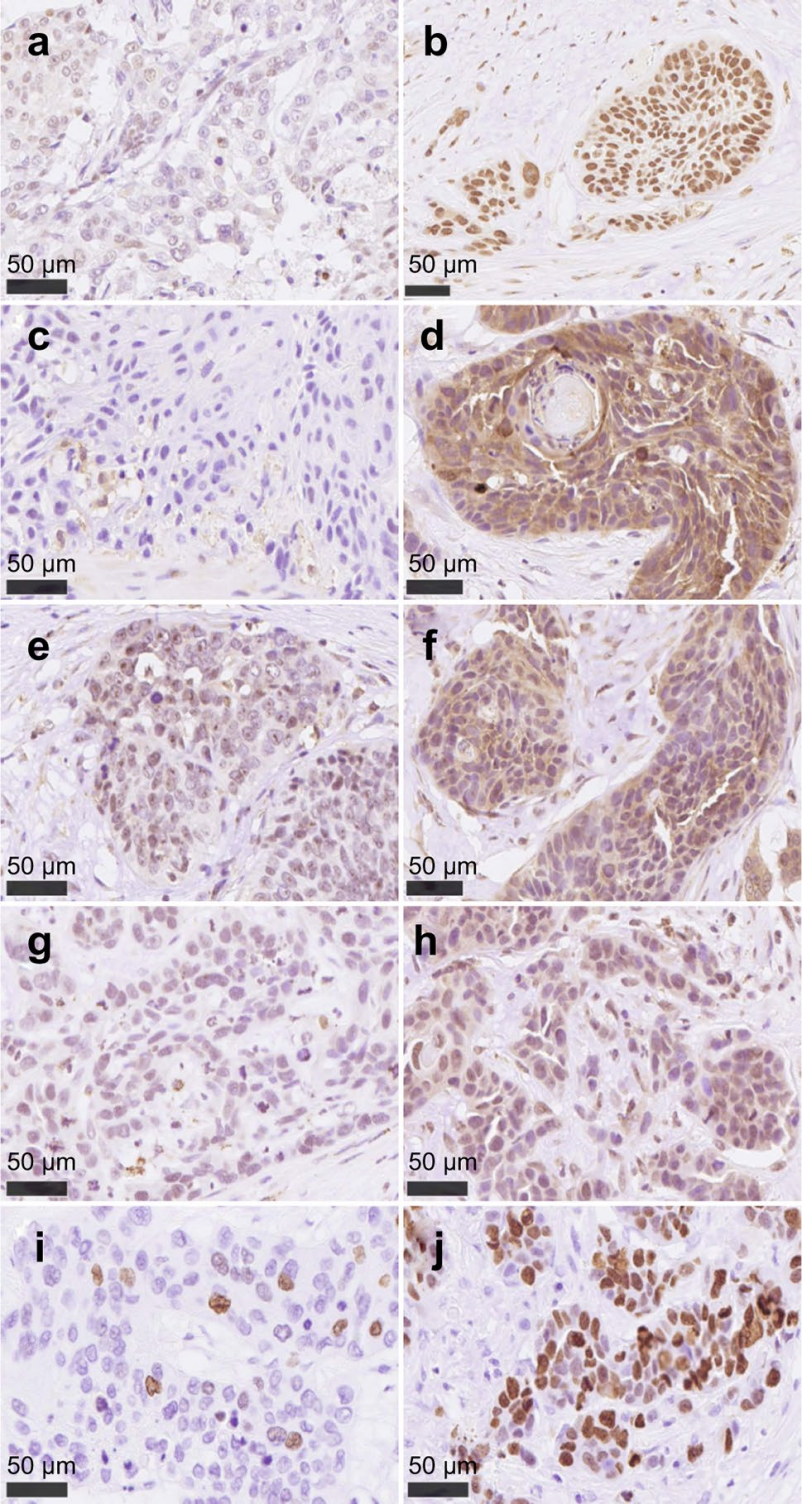
Representative illustration of immunohistochemical features. **a** Low Nrf2 expression. **b** High Nrf2 expression: representative specimen depicting immunoreactivity in the nuclei of carcinoma cells. **c** Low TXNRD1 expression. **d** High TXNRD1 expression. **e** Low HO-1 expression. **f** High HO-1 expression. **g** Low 8-OHdG levels. **h** High 8-OHdG levels: representative specimen depicting immunoreactivity in the cytoplasm of carcinoma cells. **i** Low Ki-67 levels. **j** High Ki-67 levels: representative specimen depicting immunoreactivity in the nuclei of carcinoma cells

Representative histopathological findings for Nrf2, TXNRD1, HO-1, 8-OHdG, and Ki-67 are presented in Fig. 1. A significant positive correlation was detected between Nrf2 and TXNRD1 (*P* = 0.001) and TXNRD1 and HO-1 (*P* = 0.025) (Supplementary Fig. 2). Multivariate logistic regression analysis revealed that Gender (*P* = 0.043), cT (*P* = 0.012), cN (*P* = 0.039), and HO-1 (*P* = 0.041) were independent predictive factors of histological NACRT efficacy (Table 2).

**Table 2:** Multivariate logistic regression analysis of histological NACRT efficacy in Pre-NACRT biopsy specimens

Variables		Odds ratio (95% CI)	*P*	VIF
Age	< 65 vs. 65 ≤	2.711 (0.341–21.536)	0.345	1.753
Gender	Male vs. Female	0.028 (0.001–0.894)	0.043*	3.149
cT^†^	cT1-2 vs. cT3-4	0.034 (0.002–0.473)	0.012*	2.289
cN^†^	cN0 vs. cN1-2	0.102 (0.012–0.889)	0.039*	1.333
Nrf2	Low vs. High	0.141 (0.015–1.340)	0.088	1.391
TXNRD1	Low vs. High	0.140 (0.010–1.909)	0.140	2.617
HO-1	Low vs. High	0.021 (0.001–0.860)	0.041*	2.362
8-OHdG	Low vs. High	0.991 (0.969–1.015)	0.462	1.430

*NACRT* neoadjuvant chemoradiation therapy; *VIF* variance inflation factor

*Statistical significance

^†^Tumor–node–metastasis (TNM) classification based on the 8th edition of the TNM classification of malignant tumors

### Correlation between the expression status of antioxidant proteins and clinical outcomes/prognostic factors in patients

**Fig. 2:**
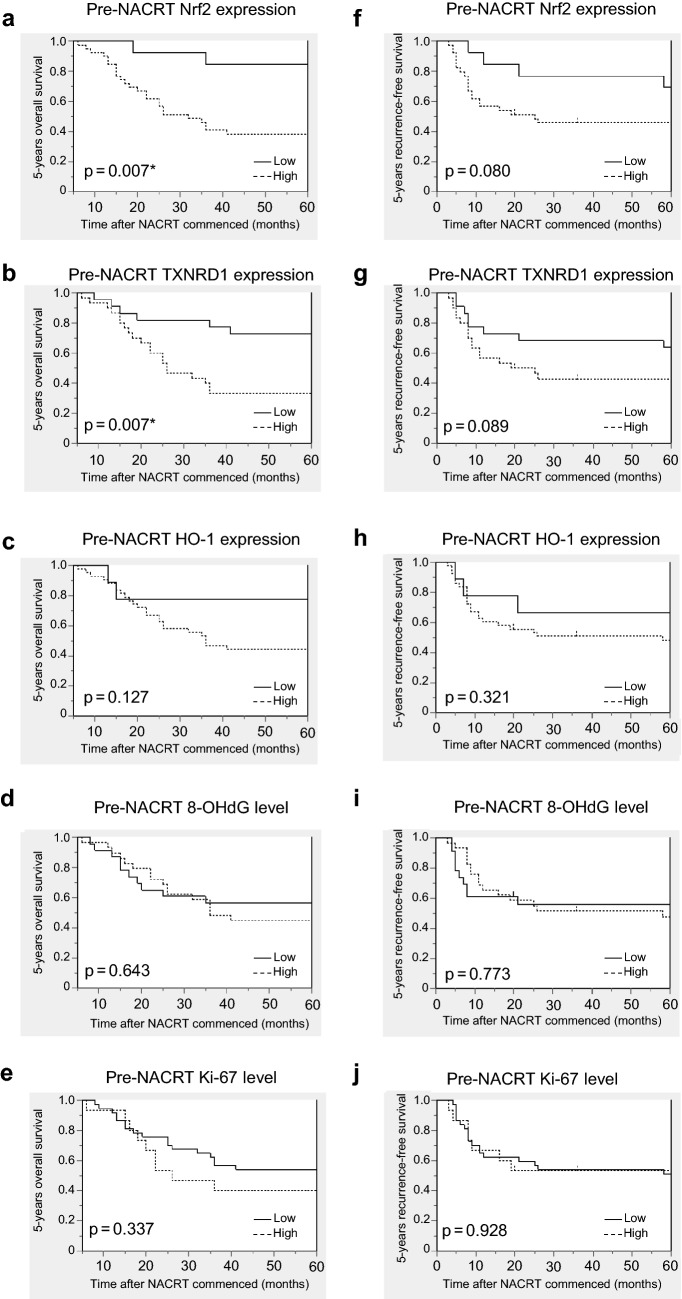
Kaplan–Meier estimates of OS and RFS based on pre-NACRT expression status of biomarkers. Kaplan–Meir estimates of OS (**a**, **b**, **c**, **d**, **e**) and RFS (**a**, **b**, **c**, **d**, **e**). The five-year OS was significantly lower in pre-NACRT specimens with high **a** Nrf2 and **b** TXNRD1 expression. *OS* overall survival; *RFS* recurrence-free survival; *NACRT* neoadjuvant chemoradiation therapy

The five-year OS rate was significantly lower in the high Nrf2 (*P* = 0.007) and TXNRD1 (*P* = 0.007) expression groups in ESCC patients (Fig. 2). No significant differences were detected in the analysis of the five-year RFS (Fig. 2). Univariate analysis revealed that the OS rate was significantly associated with high Nrf2 (*P* = 0.018) and TXNRD1 (*P* = 0.011) status (Table 3). Multivariate analysis revealed that high TXNRD1 status was the only independent prognostic factor among the variables examined in this study (*P* = 0.049) (Table 3). None of the variables examined was significantly associated with RFS (Table 3).

**Table 3:** Univariate and Multivariate analysis of 5-year OS and RFS in Pre-NACRT biopsy specimens

Variables		Univariate analysis (OS)		Multivariate analysis (OS)		VIF	Univariate analysis (RFS)	
		HR (95% CI)	*P*	HR (95% CI)	*P*		HR (95% CI)	*P*
Age	< 65 vs. 65 ≤	0.727 (0.329–1.602)	0.428	0.389 (0.149–1.015)	0.054	1.230	0.483 (0.209–1.114)	0.088
Gender	Male vs. Female	0.968 (0.365–2.568)	0.948	0.326 (0.559–5.735)	0.326	1.364	1.324 (0.531–3.301)	0.547
cT^†^	cT1-2 vs. cT3	1.442 (0.579–3.592)	0.432	1.439 (0.541–3.831)	0.466	1.135	1.357 (0.569–3.235)	0.491
cN^†^	cN0 vs. cN1-2	0.901 (0.379–2.145)	0.814	0.655 (0.242–1.771)	0.405	1.140	1.629 (0.614–4.323)	0.327
Nrf2	Low vs. High	5.754 (1.356–24.412)	0.018*	2.713 (0.523–14.067)	0.235	1.705	2.483 (0.854–7.221)	0.095
TXNRD1	Low vs. High	3.280 (1.309–8.218)	0.011*	3.354 (1.007–11.173)	0.049*	1.910	2.010 (0.872–4.635)	0.101
HO-1	Low vs. High	2.893 (0.683–12.255)	0.149	4.412 (0.724–26.902)	0.108	1.456	1.807 (0.542–6.028)	0.336
8-OHdG	Low vs. High	1.203 (0.545–2.651)	0.647	0.685 (0.253–1.855)	0.456	1.468	1.121 (0.508–2.475)	0.777
Ki-67	Low vs. High	1.477 (0.657–3.321)	0.345	0.685 (0.371–2.072)	0.764	1.301	0.961 (0.404–2.289)	0.929

*OS* overall survival; *RFS* recurrence-free survival; *NACRT* neoadjuvant chemoradiation therapy; *VIF* variance inflation factor

*Statistical significance

^†^Tumor–node–metastasis (TNM) classification based on the 8th edition of the TNM classification of malignant tumors

### Correlation between differences in expression (Δ) and NACRT resistance

**Fig. 3:**
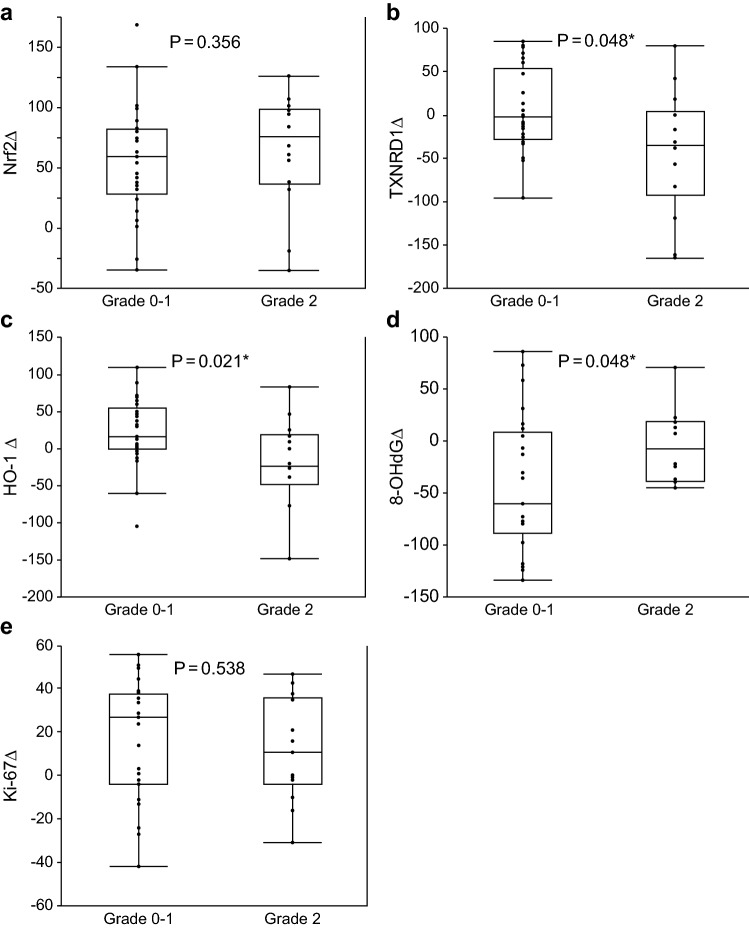
Correlation between differences in expression (Δ) and responses to NACRT. Significant differences were observed for **b** TXNRD1Δ (*P* = 0.048), **c** HO-1Δ (*P* = 0.021), and **d** 8-OHdGΔ (*P* = 0.048) between the NACRT-effective and -ineffective groups. No significant differences were observed for **a** Nrf2Δ (*P* = 0.356) and **e** Ki-67Δ (*P* = 0.538). *NACRT* neoadjuvant chemoradiation therapy

TXNRD1Δ (*P* = 0.048), HO-1Δ (*P* = 0.021), and 8-OHdGΔ (*P* = 0.048) were significantly different in the NACRT-ineffective groups compared to the NACRT-effective groups (Fig. 3).

## Discussion

First, the results of our present study revealed that HO-1 status in pre-NACRT endoscopic biopsy specimens could predict the efficacy of NACRT in the patients. In addition, TXNRD1 status in the pre-therapeutic endoscopic biopsy specimens predicted OS of the patients examined in this study. Surgical resection without NAC or NACRT could therefore facilitate curative resection in ESCC patients, who exhibited high HO-1 status, because of the frequent ineffectiveness of neoadjuvant therapy in the patients associated with aggressive biological behavior [12, 21, 22]. However, the prognosis of curative resection without preoperative therapy is certainly poor [3], which is a limitation associated with therapeutic strategies in ESCC. Further investigation of new adjuvant therapeutic strategies is necessary.

TXNRD1Δ, HO-1Δ, and 8-OhdGΔ were significantly associated with therapeutic efficacy. The significant difference in TXNRD1Δ, HO-1Δ, and 8-OHdGΔ values between the ineffective and effective groups also suggested that the ineffective group elicited a greater antioxidant response. However, no significant differences were observed in Nrf2Δ values between the two groups. These results indicate that patients in the NACRT-ineffective group exhibit an antioxidant response that involves a selective and stronger upregulation of TXNRD1 and HO-1 expression.

The present study has some limitations. First, ESCC is characterized by intertumoral heterogeneity; therefore, the endoscopic biopsy site could have considerably influenced antioxidant protein expression. Second, as carcinoma cells completely disappeared after NACRT in some cases, protein expression changes in these cells remained unevaluated. Third, the sample size of the study was rather small and may not have been sufficient to deny that the results of multivariate analysis are by chance. Further investigations with larger sample size are required to elucidate the clinicopathological significance of results of our present study.

## Conclusions

NACRT therapeutic efficacy and clinical outcomes in ESCC patients can be predicted by examining TXNRD1 and HO-1 expression status in carcinoma cells in pre-therapeutic endoscopic biopsy and surgically resected specimens. Further investigation could lead to the identification of potential prognostic factors for ESCC.

## Supplementary Information

Below is the link to the electronic supplementary material.

Supplementary file1 Supplementary Fig. 1 Diagram of excluded patients in each analysis. Among the 69 pre-therapeutic biopsy specimens, 17 were not processed at our institution, and relevant data were not available; as such, they were excluded from the analysis. Thirteen patients had minimal or no detectable residual carcinoma cells of the primary tumor in histopathological specimens, and 39 samples were available for comparison of immunoreactivity before and after NACRT. (PDF 57 KB)

Supplementary file2 Supplementary Fig. 2 Correlation between antioxidant proteins. A significant positive correlation was detected between Nrf2 and TXNRD1 (*P*  =  0.001) and TXNRD1 and HO-1 (*P*  =  0.025). No significant correlation was detected between Nrf2 and HO-1 (*P*  =  0.442). (PDF 135 KB)
