# Supplementary figures and images for: Correlation between TXNRD1/HO-1 expression and response to neoadjuvant chemoradiation therapy in patients with esophageal squamous cell carcinoma
Source: Esophagus. 2022 Jan 8;19(3):436–43. doi: 10.1007/s10388-021-00904-3 (PMC9166848; doi:10.1007/s10388-021-00904-3)

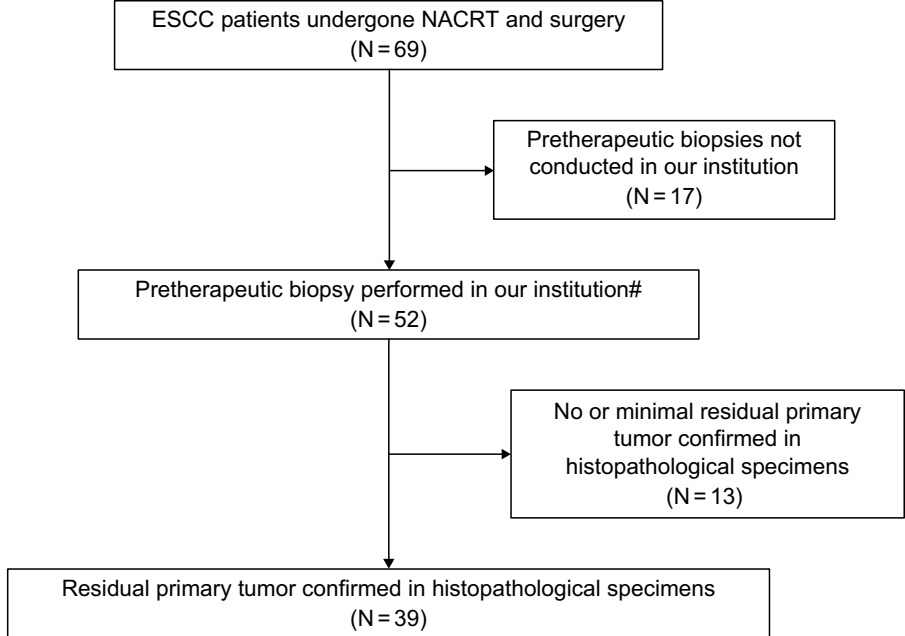

# Full analysis set

Supplement: Supplementary file 1 — Supplementary file1 Supplementary Fig. 1 Diagram of excluded patients in each analysis. Among the 69 pre-therapeutic biopsy specimens, 17 were not processed at our institution, and relevant data were not available; as such, they were excluded from the analysis. Thirteen patients had minimal or no detectable residual carcinoma cells of the primary tumor in histopathological specimens, and 39 samples were available for comparison of immunoreactivity before and after NACRT. (PDF 57 KB) [file 10388_2021_904_MOESM1_ESM.pdf]

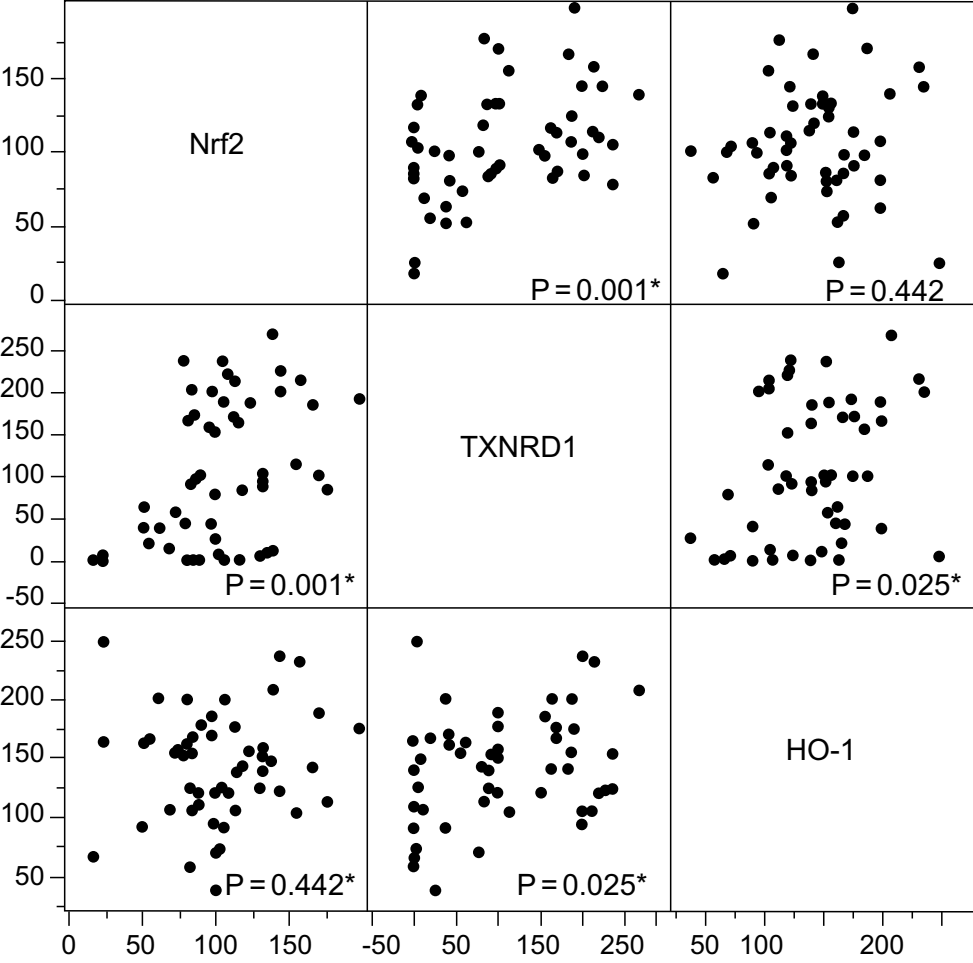

Supplement: Supplementary file 2 — Supplementary file2 Supplementary Fig. 2 Correlation between antioxidant proteins. A significant positive correlation was detected between Nrf2 and TXNRD1 (P = 0.001) and TXNRD1 and HO-1 (P = 0.025). No significant correlation was detected between Nrf2 and HO-1 (P = 0.442). (PDF 135 KB) [file 10388_2021_904_MOESM2_ESM.pdf]
